# Evidence that remdesivir treatment reduces viral titers in patients with COVID-19

**DOI:** 10.1128/aac.01266-24

**Published:** 2024-11-07

**Authors:** Simon Boyd, Shivani Singh, William H. K. Schilling, Nicholas J. White

**Affiliations:** 1 Mahidol Oxford Tropical Medicine Research Unit, Faculty of Tropical Medicine, Mahidol University, Bangkok, Thailand; 2 Centre for Tropical Medicine and Global Health, Nuffield Department of Medicine, University of Oxford, Oxford, United Kingdom; IrsiCaixa Institut de Recerca de la Sida, Barcelona, Spain

**Keywords:** coronavirus, viral load, antiviral agents

## LETTER

We respectfully disagree with the conclusion of the recent letter by Faghihi and Yan entitled “Remdesivir treatment does not reduce viral titers in patients with COVID-19” (1). In the largest and most detailed assessment of viral clearance in COVID-19 (the PLATCOV multicentre, randomized, controlled adaptive, platform trial), remdesivir accelerated the rate of oropharyngeal viral clearance by 42% compared to contemporaneous controls (2). This antiviral effect is comparable to that of molnupiravir and is second only to ritonavir-boosted nirmatrelvir of the small molecule antivirals (3). In order to characterize pharyngeal SARS-CoV-2 clearance accurately, multiple samples must be measured early in the course of the infection as there is substantial intra-subject variance in the estimated densities and natural (untreated) viral clearance is increasingly rapid (4). Participants in the PLATCOV trial are enrolled within 96 hours of symptom onset and have 18 swabs (nine duplicates) in the first 8 days. Most studies reporting viral clearance, including those cited selectively by Faghihi and Yan, have taken too few samples and are therefore highly vulnerable to type 2 errors (5). The different clinical and virological results with remdesivir reported in the medical literature are likely explained by the phase of the disease assessed (antivirals are much less effective in hospitalized patients in whom inflammatory pathology dominates), insufficient statistical power to detect clinical benefits or characterize virological responses (small sample sizes and insufficient sampling), and the declining event rates as COVID-19 becomes a milder illness. Although the interim results of the large SOLIDARITY randomized controlled trial did not show a benefit from remdesivir, the final results did show a statistically significant improvement (6), and as a consequence, the World Health Organization has changed its guidelines and now recommends remdesivir. In summary, there is abundant clinical trial evidence that remdesivir both accelerates SARS-CoV-2 clearance (7, 8) and provides clinical benefit in COVID-19.
